# Automating venous thromboembolism risk assessment: a dual-branch deep learning method using electronic medical records

**DOI:** 10.3389/fmed.2023.1237616

**Published:** 2023-08-10

**Authors:** Jianhua Yang, Jianfeng He, Hongjiang Zhang

**Affiliations:** ^1^Faculty of Information Engineering and Automation, Kunming University of Science and Technology, Kunming, China; ^2^First People's Hospital of Anning City (Jinfang Branch), Anning, China

**Keywords:** venous thromboembolism, deep learning, electronic medical record, intelligent assessment, Padua

## Abstract

**Background:**

Venous thromboembolism (VTE) is a prevalent cardiovascular disease. Although risk assessment and preventive measures are effective, manual assessment is inefficient and covers a small population in clinical practice. Hence, it is necessary to explore intelligent methods for VTE risk assessment.

**Methods:**

The Padua scale has been widely used in VTE risk assessment, and we divided its assessment into disease category judgment and comprehensive clinical information judgment according to the characteristics of the Padua scale. We proposed a dual-branch deep learning (DB-DL) assessment method. First, in the disease category branch, we propose a deep learning-based Padua disease classification model (PDCM) for determining patients' Padua disease categories by considering patients' diagnosis, symptoms, and symptom weights. In the branch of comprehensive clinical information, we use the Chinese lexical analysis (LAC) word separation technique, combined with professional corpus and rules, to extract and judge the comprehensive clinical factors in the electronic medical record (EMR).

**Results:**

We validated the accuracy of the method with the Padua assessment results of 7,690 Chinese clinical EMRs. First, our proposed method allows for a fully automated assessment, and the average time to assess one patient is only 0.37 s. Compared to the gold standard, our method has an Area Under Curve (AUC) value of 0.883, a specificity value of 0.957, and a sensitivity value of 0.816 for assessing the Padua risk patient class.

**Conclusion:**

Our DB-DL assessment method automates VTE risk assessment, thereby addressing the challenges of time-consuming evaluation and limited population coverage. Thus, this method is highly clinically valuable.

## Introduction

Venous thromboembolism (VTE) is a disease with high morbidity and a high risk of death (1). Reportedly, there are ~10 million cases of VTE annually worldwide (2), and the incidence of VTE can be as high as 0.2% per year (3). VTE has become the third leading cause of cardiovascular disease-related death (4). Effective prevention of VTE can significantly reduce its incidence, and VTE risk assessment plays a crucial role in clinical practice (5). However, only a small proportion of patients currently receive VTE prophylaxis in China (6). Therefore, the prevention and treatment of VTE are quite important in medical practice.

The current approach to VTE prevention is to assess the patient's risk level for the disease through scales and to take different preventive approaches according to the different risk levels (7). Common risk assessment scales include Padua, Caprini, and Wells. Authorities such as the American College of Chest Physicians recommend the Padua scale as a risk assessment method for VTE (8, 9). The Padua Risk Assessment Scale is designed to assess a patient's disease category and their combined clinical status, which incorporates elements such as medication use, height and weight, and surgical status. The Padua Scale utilizes a linear weighting method to obtain risk assessment scores and risk levels. The Padua scale is shown in Table 1. Compared with other scales, the Padua scale is highly accurate, relatively easy to judge, and widely applicable. However, the current process of intelligently assessing the Padua risk by doctors is time-consuming. In addition, doctors may overlook the association between the patient's disease and thrombosis, which may lead to risk assessment in which some risk factors may be neglected, thereby leaving the patient without the correct prevention protocol (10). Furthermore, assessment results can be heterogeneous due to differences in the doctors' understanding of the disease. Therefore, it is important to explore an intelligent and efficient automated assessment method for determining the Padua scale to prevent VTE.

**Table 1:** Padua scale.

**Risk factors**	**Score**
Prior VTE	3
Active cancer	3
Heart/respiratory failure	1
Acute MI/ischemic stroke	1
Acute infection/rheumatologic disorder	1
Elderly age (≥70 years)	1
BMI ≥30 kg/m^2^	1
Ongoing hormonal treatment	1
Thrombophilic	3
Reduced mobility	3
Recent (≤1 month of) trauma and/or surgery	2

Artificial intelligence can learn and extract key features from medical data to automate the analysis and processing of medical data (11). Some research has been carried out on VTE assessment using artificial intelligence techniques. However, most studies have focused on exploring risk factors for VTE and constructing various assessment scales to enhance accuracy in different patient populations (12–15). Few studies have proposed automatic risk assessment methods for VTE based on causative factors. For example, Pierre et al. (10) used International Classification of Diseases, 9th Revision (ICD-9) to match billing codes in a data warehouse for the purpose of automatically assessing the Padua scale. This approach requires building a complete enterprise data warehouse (EDW) and transforming electronic medical record (EMR) text into structured data to determine risk factors. Similarly, Qatawneh et al. (16) transformed the 35 assessment items of the Caprini scale into numerical variables and input them into a multilayer perceptron (MLP) to achieve an automatic assessment of patients' VTE risk. Chen et al. (17) designed medical text annotation for the scale items of the Wells scale and automatically assessed the Wells scale by extracting entities and relationships. Nonetheless, current research has mostly focused on exploring risk factors for constructing different assessment scales to improve accuracy in different patient populations. Although several studies have explored methods for automated VTE risk assessment, these methods have limitations, such as requiring significant manual intervention and time investment, focusing only on patient diagnosis in terms of disease category assessment, and ignoring the potential influence of clinical patient symptoms on assessment results. Few studies have examined the intelligent use of the Padua scale assessment, a widely used method in clinical internal medicine. The purpose of this study is to explore a whole-process intelligent risk assessment method based on deep learning for the Padua scale and to improve the validity and accuracy of the intelligent assessment method for practical application in VTE prevention. The study will provide a new direction for the application of deep learning technology in clinical research.

First, we used each patient's EMR as the assessment target. EMR text is an important text resource that includes a variety of information about a patient's medical process and is widely used at all levels of care (18). We can extract the factors associated with the Padua scale from the EMR and use natural language processing (NLP) techniques in deep learning for automated risk assessment.

Second, we divided the assessment of the Padua scale into two branches according to its characteristics: the disease category branch (Branch A) and the clinical comprehensive factor (Branch B). In Branch A, we proposed the Padua disease classification model (PDCM), extracted features of diagnostic and symptom texts by NLP techniques (where the information of symptoms comes from Branch B), and designed algorithms to calculate a symptom weight matrix (SWM) of different importance to increase feature information. Then, the above information is fused to determine the patient's disease category using a deep learning model. In Branch B, we used Chinese lexical analysis (LAC) (19) technology, combined with professional corpus and rules, to automatically extract and judge the comprehensive clinical factors, such as patient symptoms, surgery, medication, and activity status of the EMR, thereby solving the problem that the Padua-related factors needed to be extracted manually. In summary, this study aims to achieve complete automation of the Padua scale assessment process by constructing a two-branch method. This approach not only saves time and reduces labor costs but also considers the influence of patient symptoms when assessing disease categories, thereby improving the accuracy and precision of intelligent assessment.

## Materials and methods

### Data

In this study, we utilized data from International Classification of Diseases, 10th Revision (ICD-10) (20), ICD-9-CM3, DiseaseKG (21), and the World Health Organization's Drug List (22) to construct the proposed dual-branch deep learning (DB-DL) assessment method. For the evaluation and testing of our method, we used a dataset of EMR data from independent hospitals, which served as our gold standard. We split these data into three distinct categories: training data for the PDCM, medical corpus data, and EMR test data. These categories are elaborated further in the following sections.

#### Padua disease classification model training data (PDCM training data)

ICD-10 (20) is a library of medical terminology and corresponding codes developed by the World Health Organization, providing an authoritative and widely used classification and coding system for the medical profession. ICD-10 is widely recognized by the medical community for its broad scope of coverage, which can provide a consistent terminology and coding system for medical practitioners and facilitate information sharing and exchange between different medical institutions.

We collated the diagnostic texts of the ICD-10 according to the disease categories of the Padua scale. The number of diagnostic texts for each category is shown in Table 2. Several methods were used to process the data to better fit the clinical situation.

**Table 2:** Padua category ICD-10 diagnostic text.

**Category**	**Count**	**Symptom count**
Prior VTE	51	42
Active cancer	1,097	248
Heart/respiratory failure	27	61
Acute MI/ischemic stroke	75	51
Acute infection/ rheumatologic disorder	750	862
Others	20,007	11,726

##### Data amplification

In real-life cases, there is uncertainty in the diagnostic conclusions of doctors due to the complexity of the disease and the difficulty of diagnosis. VTE risk cannot be assessed without a clear diagnosis. To identify uncertain diagnoses among doctors, we randomly selected 10% of the Padua category diagnoses and combined frequently occurring uncertain diagnostic descriptions such as “?” and “undecided” as negative samples.

##### ICD diagnosis and symptom integration

This study used the DiseaseKG (21) data, which were sourced from an authoritative Chinese medical website. This database covers 44,656 medical terms generated during medical procedures in all aspects of medical care and provides a correspondence between 312,159 medical terms. DiseaseKG has broad coverage and is a reliable data source. We used the database to integrate the symptoms of the corresponding diagnosis and add characteristic information. Specifically, the symptoms of the disease are one of the keys to determining the category of the disease. We integrate the symptoms of the corresponding diagnosis to add characteristic information. There are 54,710 triples in DiseaseKG (21), which represent diseases and symptoms corresponding to diseases. We used the ICD-10 diagnosis text to match “Disease” in the triples to incorporate the corresponding “Symptoms” for the diagnosis. The number of symptoms corresponding to each category is shown in “Symptom Count” in Table 2.

#### Medical corpus data

Medical corpora are an important source of medical text terms that can be widely used in clinical decision-making, mortality prediction, and other clinical applications (23). We used four corpora, which consist of some standard corpora and summaries of corresponding common terms. The surgical/trauma corpus was obtained from ICD-9-CM3 (24). The hormonal drug corpus was obtained from the World Health Organization's Drug List (22). The reduced mobility corpus was derived primarily from the summary of clinical terms, such as “deep coma.” The symptom corpus comes from the symptoms in the DiseaseKG (21). Each corpus and its corpus quantity are shown in Table 3.

**Table 3:** Corpus data.

**Category**	**Count**
ICD-9-CM3	13,655
World health organization's drug list for hormonal treatment	452
Reduced mobility	34
DiseaseKG symptom	5,598

#### EMR test data

The test data used in this article were collected from the clinical EMRs of a hospital in Yunnan Province, including the characteristics of medical records, past history, past diagnosis, patient symptoms, patient complaints, examination results, doctor diagnosis, and treatment plan. The test data are used to evaluate the accuracy of our proposed method under real-world conditions. We collected 18,698 EMRs with Padua assessment results. EMR features with missing values were removed. Finally, 7,690 clinical EMRs and their corresponding Padua risk assessment items were obtained. The number of Padua scale items assessed by the doctor for EMR is shown in Table 4.

**Table 4:** EMR data.

**Category**	**Count**
Prior VTE	38
Active cancer	140
Heart/respiratory failure	361
Acute MI/ischemic stroke	421
Acute infection/rheumatologic disorder	1,334
Elderly age (≥70 years)	1,099
BMI ≥30 kg/m^2^	495
Ongoing hormonal treatment	123
Thrombophilic	12
Reduced mobility	108
Recent (≤1 month of) trauma and/or surgery	60

This study was approved by the Institutional Review Board of the First People's Hospital of Anning City. This study complied with the Declaration of Helsinki (accession number 2017YYLH035).

### Proposed method

**Figure 1:**
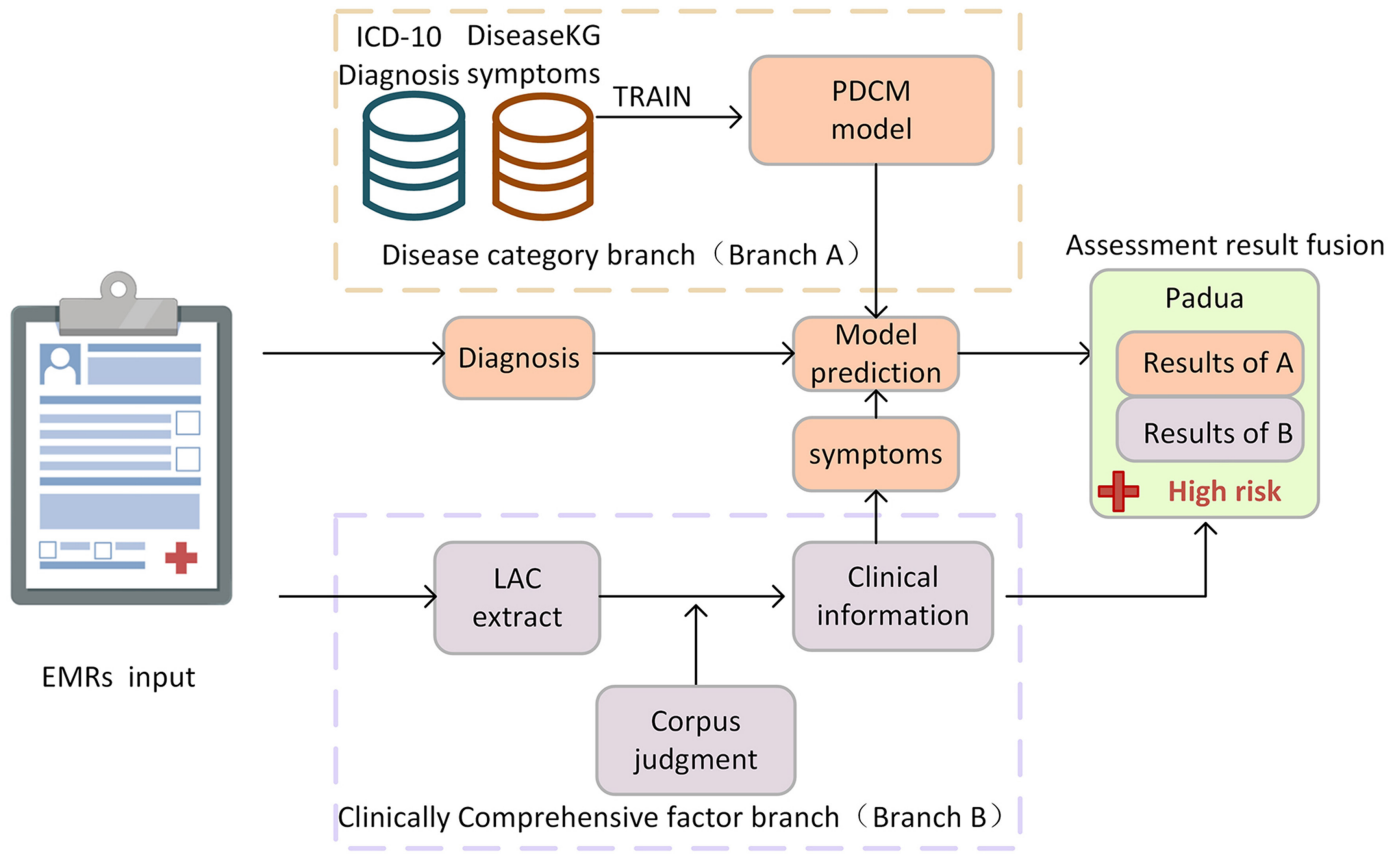
Proposed method and overall process of Padua intelligent assessment.

The doctor's diagnosis and the patient's symptoms in the EMR are key to judging the patient's Padua disease category. The combination of tests, medications, treatments, and other factors in the medical process is critical to deriving a comprehensive Padua clinical factor judgment. During the model-building phase, we propose a dual-branch method for automatic Padua assessment. In the disease category branch (Branch A), we use ICD-10 diagnosis text combined with symptom text from DiseaseKG to train a deep learning model. It is used to judge the patient's disease category items in Padua, including “active cancer,” “prior VTE,” “acute infection/rheumatic disease,” “heart/respiratory failure,” and “acute Myocardial Infarction (MI)/ischemic stroke.” In the clinical comprehensive factor branch (Branch B), we used a professional corpus to judge items including “recent (≤1 month of) trauma and/or surgery,” “reduced mobility,” and “ongoing hormone therapy” in Padua, as shown in Figure 1.

In the testing phase of our approach, we evaluate the accuracy and validity of the proposed method utilizing real EMRs, as described in Section EMR test data. For Branch A, both the patient's diagnosis and symptoms are needed. The diagnosis information from the EMR is structured and can be used directly, while symptoms are extracted by Branch B. We then use the collected diagnostic and symptom information as a unit for disease classification prediction using the PDCM. For Branch B, we utilized the LAC model to segment sentences within EMRs, and we combined a professional corpus and rules to extract and determine the information on comprehensive clinical factors, such as symptoms, activities, and medication. In this article, we further propose an automatic assessment method for “thrombophilic”, “age”, and “BMI ≥ 30 kg/m^2^ (obesity)” items of the Padua scale. Among them, age and obesity can be determined by simply extracting the corresponding data and performing calculations with a computer. According to Manderstedt et al. and Di Minno et al. (25, 26), we extracted laboratory tests for protein C, protein S, d-dimer, and antithrombin III to determine “thrombophilia”. In the following, we elaborate on the details of the methods used in both branches.

#### Padua disease category model branch (PDCM, branch A)

**Figure 2:**
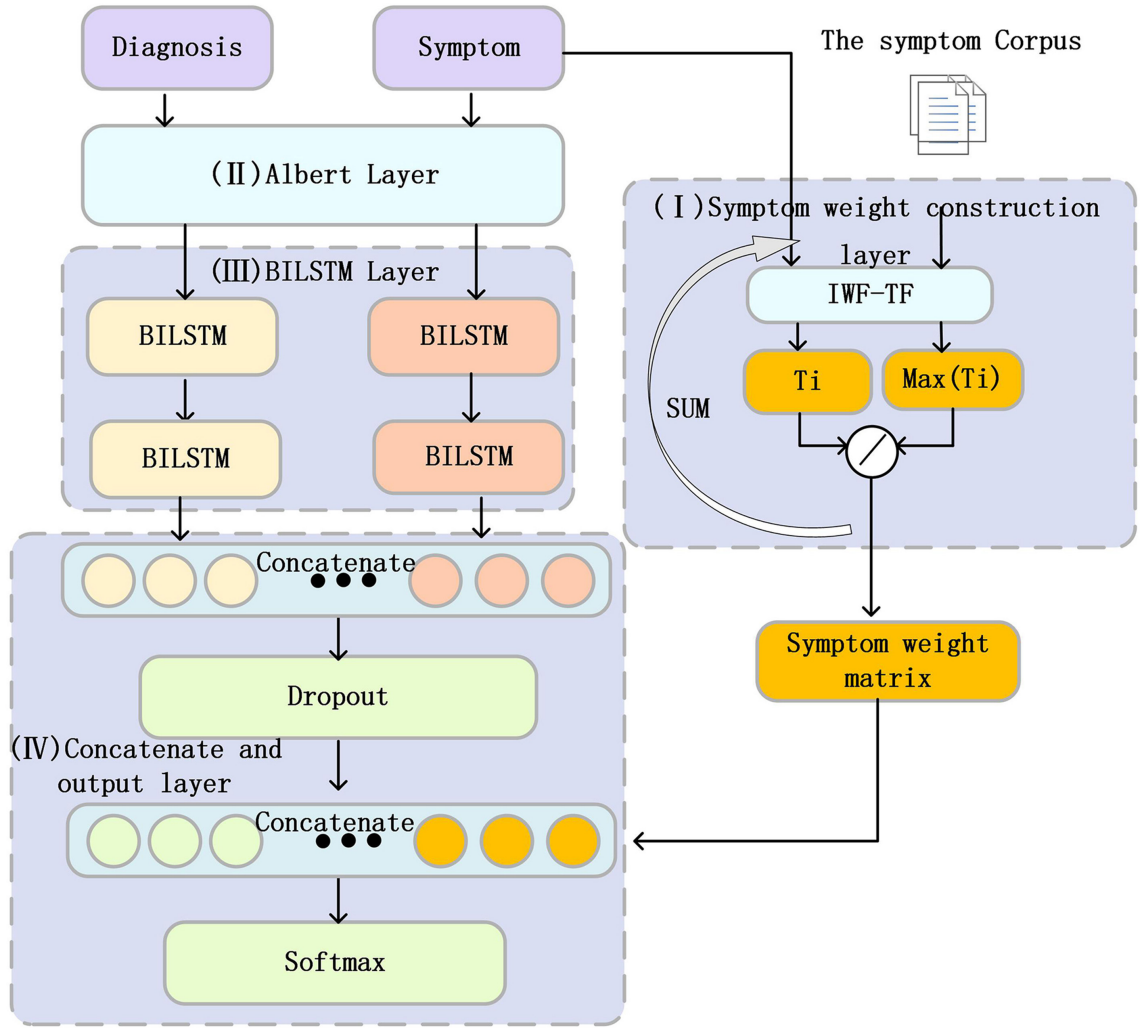
Structure of the Padua disease classification model. The Roman numerals I–IV in the figure represent the different layers in the PDCM model, which will be described in detail below.

This article proposes a classification model for Padua diseases related to the Padua scale, as shown in Figure 2. We developed an algorithm to establish a SWM layer (I) for calculating the weights of diagnoses and corresponding symptoms. Subsequently, we employed an ALBERT layer (II) to convert diagnosis and symptom texts into word vectors. Then, we used the BiLSTM layer (III) to extract features. Next, we used a concatenate and output layer (IV) to concatenate the feature information from the diagnosis and symptom texts and input them into a dropout layer to enhance our model's generalization capability. Subsequently, we concatenated the symptom weights obtained from (I) with the dropout-processed features, and finally, we predicted the merged results using a linear layer.

In summary, this study combines the ALBERT layer and the BiLSTM layer, aiming to better capture the semantic information of diagnosis and symptom text. Symptoms play a crucial role in disease diagnosis. Therefore, we incorporated the diagnosis of corresponding symptoms into our model, aiming to increase the information of the features, which in turn enhances accuracy and reliability. In addition, we propose an algorithm to calculate the SWM of different symptoms for each disease category in Padua. This SWM is fused with the information on diagnostic and symptom features extracted by BiLSTM. This allows symptoms of different importance to exert different effects on disease classification and expands the range of features. The following section provides a detailed description of each component of the PDCM.

##### Symptom weight matrix layer (I)

Some symptoms are common in various diseases (e.g., “fever”). These symptoms can easily cause noise in the classification task. The various symptoms that correspond to each diagnosis have different levels of importance to the diagnosis, while symptoms of the same category of diseases are similar. Term frequency–inverse word frequency (TF-IWF) is an algorithm used to evaluate the extent to which a word can reflect its corpus. We use the TF-IWF (27) algorithm to calculate the importance of symptoms in disease categories. The SWM was proposed according to the corresponding symptoms of the diagnosis. The SWM is calculated as follows:

In Table 2, we aggregate the symptom corpus *N*_*i*_ corresponding to the diagnosis of each category of Padua, where *N*_*i*_ represents the symptom corpus of category *i*. The total number of occurrences of a certain symptom *t* in the symptom library *N*_*i*_ is *N*_*i,t*_, and the total number of words in the symptom library *N*_*i*_ is count (*N*_*i*_). Then, the TF of symptom *t* relative to the symptom library *N*_*i*_ is as follows:


$$\begin{array}{l} TF=\frac{N_{i,t}}{count(N_{i})} \end{array}$$

Then, all *N*_*i*_ is the total symptom corpus *w*. Let the frequency of all symptoms be *W*_*c*_, among which the frequency of symptom *t* in all words of *W* is *W*_*c,t*_; then, the IWF of symptom *t* relative to the total symptom corpus is as follows:


$$\begin{array}{l} IWF=\log\frac{W_{c}}{W_{c,t}}\; \end{array}$$

Thus, TF–IWF of symptom *t* relative to *N*_*i*_ is as follows:


$$\begin{array}{l} TI=TF-IWF=TF\times IWF \end{array}$$

Finally, we developed the SWM algorithm to calculate the SWM for adding feature information. The SWM construction algorithm is shown in Table 5.

**Table 5:** Algorithm: medical symptom weight matrix construction procedure.

**Algorithm 1: medical symptom TF-IWF construct Procedure**
Input:**N**_**k**_={**N**_**0**_,**N**_**1**_,**N**_**2**_,**N**_**3**_,**N**_**4**_,**N**_**5**_},**N**_**k**_, represents Padua's symptom for different disease categories. **X**_**t**_={t1,t2...,Tn}, **X**_**t**_ are the different symptoms corresponding to the diagnosis.
Output: The constructed symptom weighting matrix.
1: ## Construct an initial one-dimensional matrix of length 6, corresponding to the 6 disease categories of the Padua scale.
2:SWM=[0,0,0,0,0,0]
3: ## Calculate the weights of the corresponding category for each symptom separately.
4: for *t* in **X**_**t**_ do:
5: for *i* in **range(k)** do:
5: If *i* in **N**_**k**_ do:
7: SWM_*k*_=(**[TI**_**i, t**_**/max(TI**_**i**_**)**])
8: ## symptom weight matrix summation for each symptom.
9: SWM = +SWM_*k*_
10: return SWM

##### ALBERT word embedding layer (II)

The initial phase of model training involves text vectorization. Currently, several vectorization options exist, including Word2vec (28), BERT (29), and ALBERT (30). BERT has emerged as the most prevalent pretraining model due to its transformer structure. It employs bidirectional encoding, which offers more robust feature extraction capabilities than Word2vec. Furthermore, BERT addresses the contextual ambiguity of words that Word2vec struggles to resolve. Recently, in 2020, Lan et al. proposed a lightweight pretraining model named ALBERT. This model simplifies BERT using decomposition embedding parameterization, cross-layer parameter sharing, and other methods that significantly reduce computational parameters. Models with few computational parameters can greatly reduce memory overhead in terms of deployment.

According to the diagnosis and length of symptoms, we use ALBERT to vectorize the input diagnostic or symptom text into vectors of size (20, 768) or (50, 768).

##### BiLSTM layer (La)

The long short-term memory (LSTM) neural network (31) is a recurrent neural network (RNN) that overcomes the gradient explosion problem of traditional RNNs. However, LSTM considers only past information and ignores future information. To use context information effectively, BiLSTM combines forward and backward LSTM is used to obtain two separate hidden states: $h_{t}^{{}^{\prime }},h_{t}$. The two hidden states are then concatenated to form the final output $h_{t}^{\sim }$ =[$h_{t}^{{}^{\prime }}$,*h*_*t*_] of time *t*.

We used two BiLSTM layers. The output dimension of the first layer of BiLSTM is (20,768) or (50,768), which aims to extract the features of the word vector, and the second layer of BiLSTM outputs the hidden state, which contains all time steps with a feature dimension of 768.

##### Concatenate and output layer (IV)

Diagnosis and symptoms were represented by BiLSTM extraction features as *h*_*diagnosis*_ and *h*_*symptoms*_, respectively. We concatenated *h*_*diagnosis*_ and *h*_*symptoms*_, *h*_*Concatenated*_ as follows:


$$\begin{array}{l} h_{Concatenated}=[h_{diagnosis}\;,h_{symptoms}] \end{array}$$

*h*_*Concatenated*_ has the features of diagnosis and diagnosis of corresponding symptoms. Then we input *h*_*Concatenated*_ to the dropout layer to increase the generalization performance of the neural network. The output of the dropout layer is represented as *h*_*Dropouted*_. Then, we concatenate the output SWM of the symptom weight matrix with *h*_*Dropouted*_ to obtain the following:


$$\begin{array}{l} h_{SWM}=[SWM\;,h_{Dropouted}]\; \end{array}$$

Finally, we input *h*_*SWM*_ to the classification layer for classification using softmax. Due to the unbalanced diagnosis of Padua's corresponding categories, we used the focal loss (32) function.

#### Clinical comprehensive factor branch (branch B)

**Figure 3:**
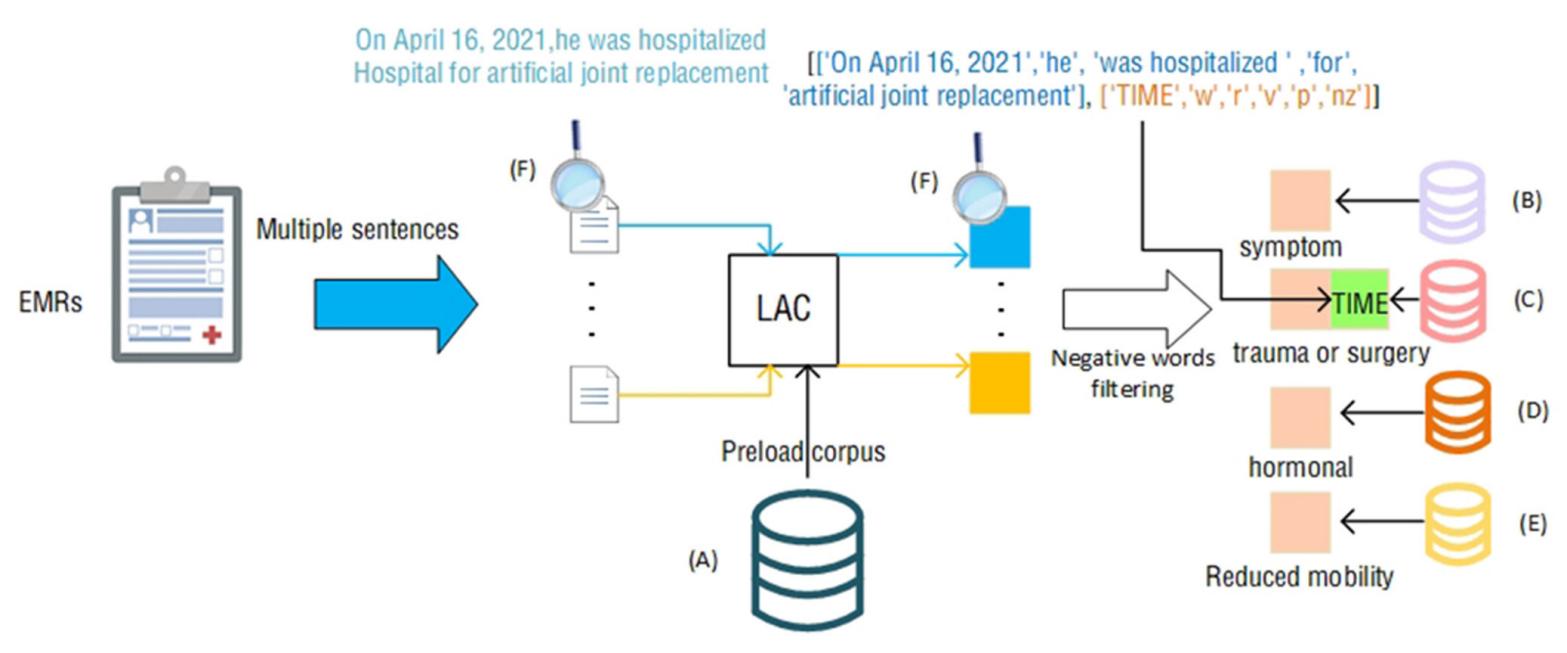
Clinically Comprehensive factor extraction judgment process. The icons next to the letters **(A–F)** represent the following contents: **(A)** total corpus; **(B)** symptom corpus; **(C)** trauma or surgery corpus; **(D)** hormone corpus; **(E)** activity reduction corpus; **(F)** process. The example content in the module, the example in the figure, is the statement “On April 16, 2021, he was hospitalized Hospital for artificial joint replacement” that appears in the EMR.

In this article, we propose a clinically comprehensive factor branch related to the Padua scale that utilizes LAC splitting techniques and negative word filtering to achieve the extraction of patient symptoms, medication information, and activity in the EMR text, as shown in Figure 3.

The LAC (19) lexical segmentation tool can perform automatic lexical segmentation of sentences and provide lexical information of words after lexical segmentation. Medical terms tend to be more accurate, and direct use of LAC lexical segmentation is likely to result in inaccurate lexical segmentation. LAC provides a method of loading an intervening lexicon that allows LAC to perform accurate lexical segmentation when accurate medical terms are encountered.

First, to accurately and precisely segment and match medical terms and diagnoses in the EMR, we used the LAC word-splitting tool. Using the total corpus as a preloaded corpus, we represent the EMR as a collection containing multiple sentences. Then, we obtained the result of LAC segmentation by feeding each sentence into the LAC model, which includes the segmented words and the corresponding lexical properties.

When we use the LAC segmentation tool for each sentence in the EMR, we can obtain a set of vocabulary and corresponding lexical properties. These vocabularies and lexical properties are the basis for performing medical terminology matching. In Section Medical corpus data, four professional corpora were selected as references for matching terms, including ICD-9-CM3, DiseaseKG-Symptoms, Reduced Mobility, and World Health Organization's Drug List. These four corpora are standard classification systems widely adopted in the medical field, and thus, they cover most of the medical terms and disease diagnostic results.

To make the matched results more accurate and reflect the actual situation of patients, we introduced a negative word matching and filtering mechanism. In medical terminology and disease diagnostic results, a negative situation often refers to the exclusion of certain symptoms or conditions. For example, “patient is not using hormones” means that the patient is not using hormones. If we were to match that description directly, the result would be “hormone use,” which does not match the patient's actual condition. Therefore, we added negative words to the matching process. When a negative word appears in the LAC result, we filter out the sentence and exclude the corresponding symptom or condition from the sentence. Negative words include but are not limited to “not used,” “not seen,” “none,” “not found,” and so on. Specifically, we extracted all the negative words and matched them according to their preceding and following contexts. For example, in the phrase “no hormone use,” “no use” is a negative word, so we filter the phrase. Similarly, in “no abnormalities seen,” “no abnormalities seen” is a negative word, and it is followed by “abnormalities,” so we will exclude “abnormalities” from the results.

By using the negative word matching and filtering mechanisms, we were able to more accurately extract the symptoms and conditions that represent the actual condition of the patient in each sentence. This has important implications for disease diagnosis and treatment, providing clinicians with a more accurate reference base, as well as providing more accurate data support for medical research. Notably, the item “recent (≤1 month of) trauma and/or surgery” has a time judgment requirement. Combined with the feature of LAC to divide sentences according to lexicality, we compare the time corresponding to the time adverb extracted by LAC with the current time to judge this item. In Figure 3, the sentence ‘On April 16, 2021, he was hospitalized Hospital for artificial joint replacement' within the Electronic Medical Record (EMR) identifies ‘On April 16, 2021' as the ‘TIME'. We can use this time and the current time to make a judgment.

In summary, our method is based on the LAC word division tool and several professional corpora to achieve accurate extraction and recognition of medical terms and disease diagnosis results in EMRs through matching and filtering mechanisms.

## Results

### Evaluation index and experimental environment

In this article, the AUC (33) and F1 (34) were selected as the main evaluation indexes. In addition, three common multi-label classification evaluation indexes are used as subevaluation indexes, including the Hamming Distance (HD) (35), One-Error (OE) (36), Label Ranking Loss (RL) (37), and Coverage (Cov) (38). The higher the AUC and F1 values are, the lower the HD, OE, and Cov values are, and the better the performance of the model.

For the purposes of training, executing, and evaluating performance, the training experiment was executed on a computer running the Windows 10 operating system with an Intel^®^ Core™ i7-11700KF CPU, an NVIDIA GTX3080 graphics processing unit, and 10 GB of memory. The computer was equipped with 32 GB of RAM and ran in the Python 3.7 and TensorFlow 2.7.0 environments.

Our proposed approach is divided into a deep learning model for disease diagnosis and an assessment of clinical situations. We evaluated the two parts of the experiments separately.

### Experiment of branch A

The items “active cancer,” “prior VTE,” “acute infection/rheumatologic disorder,” “heart/respiratory failure,” and “acute MI/ischemic stroke” are based on the proposed PDCM.

We split the PDCM training data in Section Padua disease classification model training data (PDCM training data) by 7:3 as a training set and a validation set. The number of training iterations was 100, and the model performance was checked using the validation set. We used the early-stopping (39) method during the training. If the effect has not improved for 10 consecutive rounds, then training is terminated. The previous model with the highest F1 was saved.

#### Model comparison results and analysis

We test our proposed method using the EMR data in Section EMR test data. To further evaluate the effectiveness of our proposed PDCM, we have selected the following baseline models for comparison: IDCNN (40), BiRNN (41), Transformer (42), TEXTCNN (43), and BiLSTM (44) are commonly used in the field of text classification for comparison. Among the comparative methods, we also use diagnostic and symptom text inputs. We chose ALBERT as our vectorization technique for both PDCM and the comparative methods because of its extensive embedding features, lower parameter count, and suitability for clinical deployment, as described in Section Ablation experiment. Below, we present the results in Table 6. Furthermore, in the following section, we provide a detailed description of the prediction process for each approach.

**Table 6:** Comparative experimental results.

**Category**	**AUC**	**HD**	**RL**	**Cov**	**OE**	**F1**
TEXTCNN	0.700	0.047	0.132	0.349	0.223	0.722
BiRNN	0.735	0.049	0.151	0.404	0.225	0.769
IDCNN	0.769	0.052	0.135	0.360	0.234	0.752
Transformer	0.779	0.044	0.145	0.381	0.197	0.791
BiLSTM	0.791	0.039	0.117	0.390	0.179	0.800
PDCM (Ours)	0.836	0.036	0.100	0.282	0.167	0.838

##### TEXTCNN

TEXTCNN uses convolutional neural networks for text classification. In TEXTCNN, we employ ALBERT for vectorizing diagnostic and symptom text data. Following the original paper, we use convolutional kernels of sizes 3, 4, and 5 to capture features from the text. Max pooling is applied to extract the most salient features produced by each convolutional kernel. The pooled outputs are then merged and combined with the feature information from the diagnosis and symptoms. Finally, the merged features are passed through a dropout layer and a classification layer to predict the disease category of the patients. Compared with TEXTCNN, our method improves the AUC by 13.6%.

##### BiRNN

BiRNN is an RNN model that can input information in both forward and backward transmission directions. In BiRNN, we input the vectorized diagnostic and symptom information separately into BiRNN as time steps. This enables us to extract text features by first obtaining the features of each time step. Subsequently, we input the time step features into the next BiRNN to obtain the final hidden state and extract overall features. Finally, we merge the extracted diagnostic and symptom features, apply a dropout layer, and perform classification using a linear layer. In contrast, the AUC of our method improved by 10.1%.

##### IDCNN

IDCNN introduces the concept of dilation rate, allowing the model to increase its receptive field without adding parameters. This enables capturing longer-range dependencies. In the prediction process of IDCNN, iterative dilation convolution is used to capture contextual feature information at different scales from vectorized diagnostic and symptom data. The diagnostic and symptom features are then merged after applying Dropout for regularization. Finally, the merged features are passed through the classification layer to predict disease categories. IDCNN is 6.7% less effective than PDCM (Ours) in the AUC.

##### Transformer

The structure of the Transformer is composed of an encoder and a decoder. Inputs are provided to the encoder layer, comprising vectors and positional information for diagnoses and symptoms separately. It employs stacked self-attention mechanisms and encoder–decoder attention mechanisms to capture sequence correlation information. Finally, the features of diagnoses and symptoms are concatenated, and the patient's disease category is produced through a linear layer. In contrast, the AUC of our method improved by 5.7%.

##### BiLSTM

BiLSTM is an improvement of BIRNN, which solves the problem of gradient explosion in RNN well by designing a forget gate. We vectorize the diagnosis and symptom information and input it into two BiLSTMs to extract temporal and global features, respectively. Finally, we concatenate these features, apply a dropout layer for regularization, and use a linear layer to predict the disease category of the patient. Compared with BiLSTM, our method improves the AUC by 4.5%.

##### PDCM (Ours)

Compared to BiLSTM, our PDCM model incorporates a SWM, enabling better calculation of the influence weight for each symptom category on the overall category. PDCM strengthens the association between symptoms and disease categories using a symptom weighting matrix. Additionally, PDCM expands the feature range to achieve optimal performance.

It can be seen from the data in Table 6 that the PDCM used in this article achieved the best results in terms of AUC and F1.

#### Ablation experiment

In Section Model comparison results and analysis, we experimentally concluded that the PDCM model works best. For this, we designed ablation experiments to determine the effectiveness of several methods. In the proposed method, Diagnose (Only), Diagnose + symptom, PDCM, and PDCM (Without) were used. Diagnose (Only) means training with diagnosis only, Diagnose + symptom means training with diagnosis and corresponding symptoms for diagnosis; the PDCM represents the use of diagnosis + symptom prediction while incorporating the symptom weighting matrix presented in Section Symptom weight matrix layer (I). To analyze the effectiveness of these methods, an ablation experiment was designed. In Section Padua disease classification model training data (PDCM training data), we mentioned data augmentation for the diagnostic incorporation of uncertain text. To verify the effect of this part, we removed this part of the augmentation as PDCM (Without). The experimental results are shown in Table 7.

**Table 7:** Ablation experiment.

**Category**	**AUC**	**HD**	**RL**	**Cov**	**OE**	**F1**
PDCM (Without)	0.709	0.046	0.170	0.451	0.216	0.745
Diagnose (Only)	0.782	0.041	0.140	0.389	0.190	0.798
Diagnose + symptom	0.791	0.039	0.117	0.390	0.179	0.800
PDCM (Ours)	0.836	0.036	0.100	0.282	0.167	0.838

It can be seen from the experimental results that the PDCM proposed by us has achieved the best effect. Compared with PDCM (Without), F1 and AUC were improved by 9.3 and 12.7%, respectively. We compared Diagnose (Only) with Diagnose + symptom and found a 0.9% increase in AUC after incorporation of symptoms.

**Figure 4:**
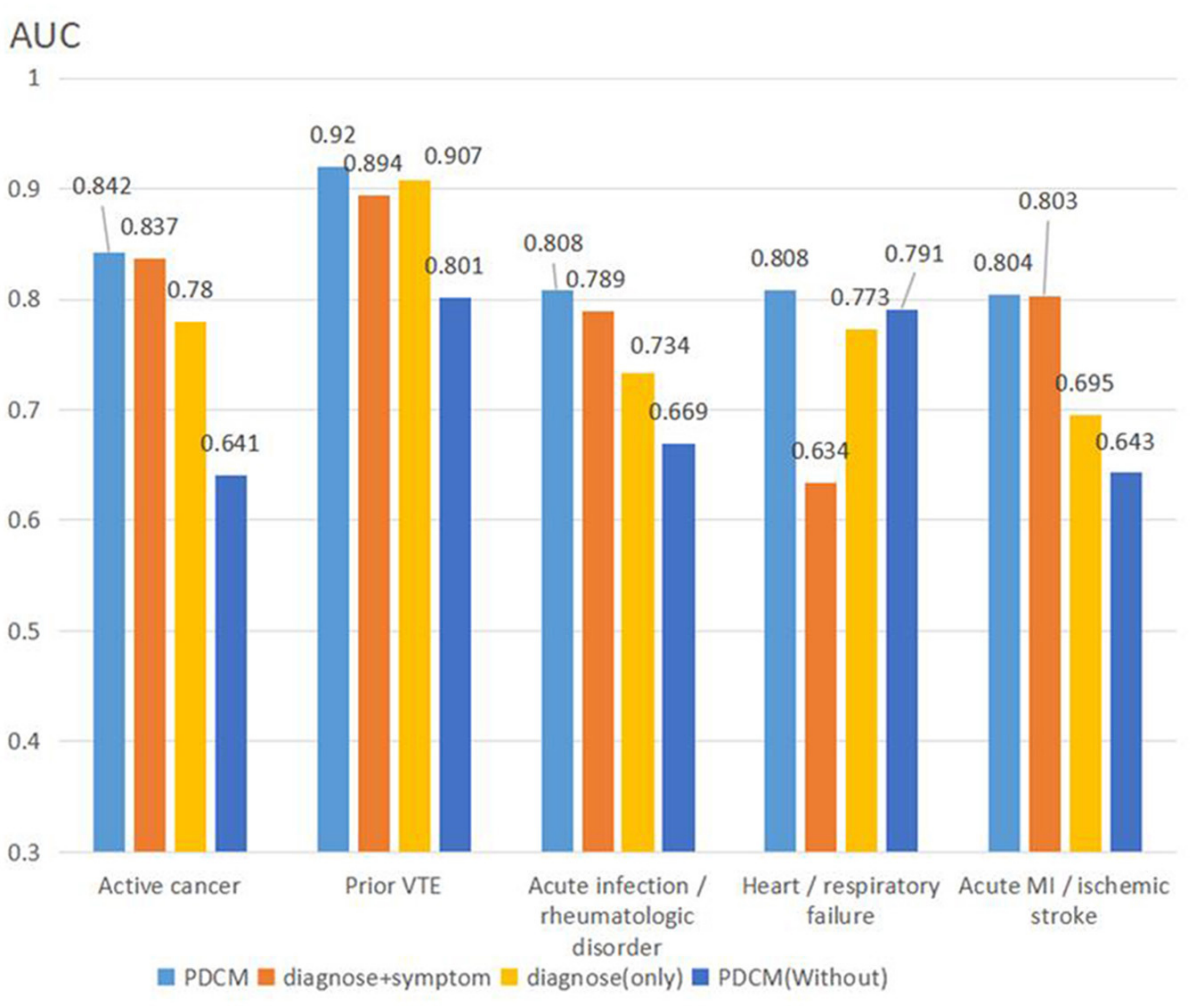
AUC values of multiple methods for ablation experiments.

To compare the differences among different methods in detail, we calculated the AUC value of each algorithm for each item, and the results are shown in Figure 4.

In summary, the PDCM proposed by us achieved the best effect. First, we compared Diagnose (Only) with Diagnose + symptom, and found that the test result was significantly higher than that of Diagnose (Only) after the integration of symptoms. The Diagnose + symptom method achieves a better effect on the evaluation of the “active cancer” item (AUC: 0.837 vs. 0.824) and the “acute infection and/or rheumatologic disorder” item (AUC: 0.789 vs. 0.749). Comparing PDCM and PDCM (Without), we found that the model effect was significantly improved in “active cancer” (AUC: 0.842 vs. 0.641), “acute infection and/or rheumatologic disorder” (AUC: 0.808 vs. 0.791), and “acute MI and/or ischemic stroke” (AUC: 0.804 vs. 0.643) items.

### Experiment of branch B (clinical comprehensive factor extraction judgment results)

**Figure 5:**
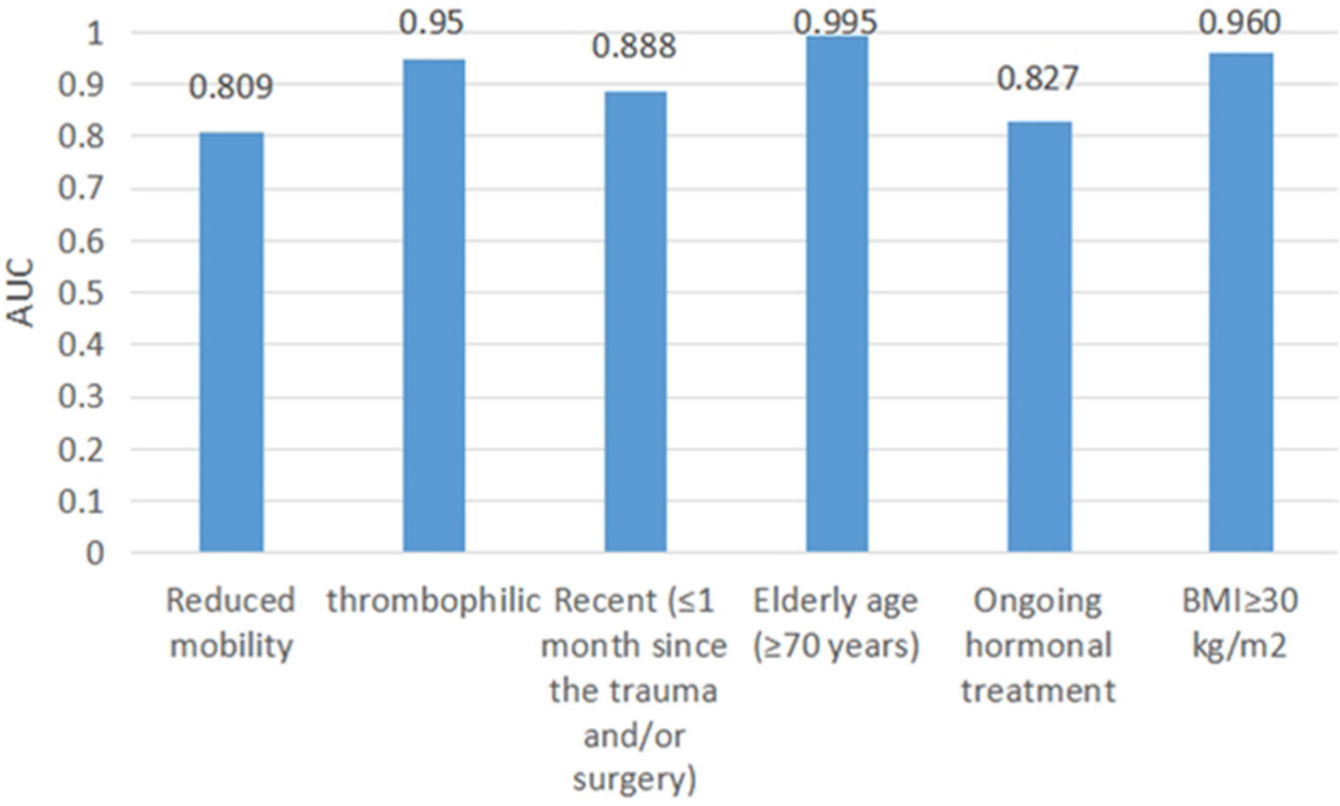
Clinical comprehensive factor branch AUC.

We test our proposed method using the EMR data in Section EMR test data. Items of “Reduced mobility,” “Recent (≤1 month of) trauma and/or surgery,” and “Ongoing treatment” use the method in Branch B. Items of “Elderly age (≥70 years),” “BMI>30 kg/m^2^,” and “thrombophilic” use computerized numerical calculations. We treated these items as independent dichotomous items using the AUC assessment. The AUC values of these items are shown in Figure 5.

Items of “Reduced mobility,” “Thrombophilic,” “Ongoing treatment,” “Recent (≤1 month of) trauma and/or surgery,” “Elderly age (≥70 years),” and “BMI>30 kg/m^2^” had AUCs of 0.809, 0.95, 0.888, 0.995, 0.827, and 0.960, respectively.

### Padua overall evaluation results

We integrated Branch A and B, presented in Section Proposed method, into the VTE software system for practical use. The scoring interface in real-world applications is shown in Figure 1. According to the authoritative standards of the American College of Chest Physicians, a total score below 3 on the Padua scale is considered low risk, and a score above 3 is considered high risk. The risk assessment level is crucial as it directly determines the patient's follow-up treatment plan. We used the scores to assess the patient's level of risk. It is a common phenomenon in healthcare that there are far fewer high-risk people than low-risk people. In this section, we use AUC, sensitivity, specificity (33), and precision value (34) to evaluate the performance of our method, which is not affected by data imbalance.

Of the 7,690 EMRs tested, 7,548 were assessed by doctors as low risk and 142 as high risk. Among the 7,548 low-risk EMRs, 4,341 samples with a score of 0 were also assessed as low risk. Although a score of 0 is also considered low risk, it represents a much lower level of risk. Considering the imbalance in the samples, we utilized the AUC value to evaluate the risk levels for 0 scores, low risk, and high risk. The AUC of 0.883 in assessing patients' risk of VTE indicates that the model has a high level of accuracy in distinguishing between low-risk and high-risk EMRs.

The precision value can be used to further evaluate the false rate of our proposed method. With an evaluation value for precision of 0.87, it indicates that our model has high precision in its predictions, denoting a low overall false positive rate and validating the reliability of our method.

We further use sensitivity and specificity to evaluate the accuracy of low and high risk. Both indicators are equally unaffected by the data imbalance. Sensitivity and specificity represent the ability of the method to detect positive samples and exclude negative samples, respectively. The high specificity value of 0.957 suggests that the model is able to correctly assess a large proportion of low-risk EMRs, while the sensitivity value of 0.816 indicates that the model is also effective at assessing high-risk EMRs.

## Discussion

Our proposed two-branch model automatically predicts VTE risk from the EMRs without doctor input, greatly reducing the burden on doctors. Currently, most patients do not receive effective VTE risk assessment and prevention (6). Our proposed method for automatic VTE risk assessment helps to improve the prevention rate of VTE. Furthermore, intelligent assessment using an artificial intelligence approach helps to eliminate the heterogeneity caused by the assessment of different doctors. Moreover, our proposed DB-DL assessment method achieves higher accuracy than other intelligent methods.

In terms of automation of VTE risk assessment, although both Pierre et al. (10) and Qatawneh et al. (16) automate the assessment to some extent, their methods require additional steps. Specifically, Qatawneh et al.'s approach involves information that primarily exists in textual form, which requires considerable time investment in converting textual information into numerical values. On the other hand, Pierre et al. require the construction of a complete EDW and subsequent transformation of EMR text into structured data to determine risk factors, which may not be feasible for healthcare organizations without available resources for building a large-scale structured data warehouse. In contrast, the object of our proposed DB-DL assessment method is EMR text, and EMR systems are widely used at all levels of healthcare systems, which makes our method more applicable. Furthermore, we elaborate on our DB-DL assessment method in Section Proposed method. The DB-DL assessment method is divided into two branches, with Branch A using our designed PDCM deep learning model to determine the patient's disease category, which mainly utilizes diagnostic and symptom text data. By using LAC combined with negative word filtering in Branch B, we are able to extract and determine the patient's symptoms, hormone use, and activity, thus providing automation of the entire DB-DL assessment method. Our method automates the entire process of assessment without manual transformation or doctor input compared to previous work.

The accuracy of the automated assessment of high- and low-risk levels in Padua is crucial and determines the next preventive measure or treatment for the patient. In terms of the accuracy of the automated assessment of the Padua scale, we tested the accuracy of our proposed DB-DL assessment method using the EMR test data as an independent data source. Pierre et al. (10), who also studied the automated assessment method regarding the Padua scale, had an AUC of 0.81 in assessing Padua high and low risk for them, while our DB (Pierre et al.) used a structured medical record from a processed data warehouse matched to ICD-9 codes to determine the patient's VTE risk items. This has less characteristic information and ignores the impact of the patient's symptoms and test results on the disease category. Such characteristics are less informative and ignore the impact of the patient's symptoms and test results on the disease category. For example, the symptom “Precardial pain” is correlated (45) with the item “Acute MI/ischemic stroke” in the Padua scale. Our DB-DL assessment method utilizes a deep learning model, PDCM, and a combination of clinical factors to determine the branch; our method also accounts for the patient's diagnosis and multiple texts, including physical examination, tests, medications, and diagnosis, to achieve higher accuracy.

In our DB-DL assessment method, Branches A and B represent different items of the Padua scale, respectively. We further discuss the accuracy of the table items represented by Branches A and B. In Branch A, we propose the PDCM deep learning model and input the patient's diagnosis + symptom prediction to determine their disease category. We compare the accuracy of the proposed PDCM model with the common disease classification deep learning models IDCNN (40), BiRNN (41), Transformer (42), TEXTCNN (43), and BiLSTM (44) on this task. BiRNN is suitable for handling sequential problems but prone to the gradient explosion problem, and our proposed method outperforms BiRNN (AUC: 0.837 vs. 0.735). The Transformer model is not affected by the gradient explosion problem of traditional RNN and can better capture relationships and dependencies at a distance in the input sequence. However, it sacrifices traditional RNN and local feature capture. In contrast, our model outperforms the Transformer model (AUC: 0.837 vs. 0.779). While IDCNN and TEXTCNN have their advantages, IDCNN may lose information, while TEXTCNN has a fixed window size that limits its ability to incorporate all textual information. Our model outperforms the IDCNN and TEXTCNN models (AUC: 0.837 vs. 0.769 and AUC: 0.837 vs. 0.700, respectively). BiLSTM can model stacked LSTM layers and better incorporate context but still falls short of our PDCM model (AUC: 0.837 vs. 0.791). The latter accounts for symptom weights and achieves higher accuracy.

To further validate the impact of each module of our proposed PDCM model on the accuracy of Branch A assessment, in Section Ablation experiment, we designed ablation experiments in which we compared four methods, namely Diagnose (Only), Diagnose + symptom, PDCM, and PDCM (Without). Diagnose (Only) represents only Diagnose + symptom stands for training and predicting patients' disease categories using Diagnose only. Diagnose + symptom stands for training and predicting patients' disease categories using Diagnose + symptom. PDCM is our proposed deep learning model, which stands for training and predicting patients' disease categories using Diagnose + symptom and fused symptom weights. In Section Padua disease classification model training data (PDCM training data), we performed Data amplification to incorporate uncertain diagnostic descriptions to improve the model's generalization performance. To analyze the effectiveness of data amplification, we removed this part of the data extension and used PDCM (Without) representation. First, we compared Diagnose (Only) and Diagnose + symptom in terms of the validity of diagnostic integration of symptoms and found that the test results after the integration of symptoms were significantly higher than those of Diagnose (Only). This indicates that the model obtained more feature information after incorporating symptom information and obtained better generalization performance in the actual test. In terms of data augmentation, PDCM (Without) and PDCM were compared. We found that many uncertain diagnoses were incorrectly judged as true by the model when no data augmentation method was used. Diagnose + symptom or PDCM can be used to verify the validity of incorporating the symptom weighting matrix in PDCM. We then compared Diagnose + symptom with the PDCM model and found that the accuracy of determining patient disease categories was substantially improved after incorporating symptom weights. The PDCM used the TF-IWF algorithm to calculate the weight of each symptom category's influence on the category and showed the best results by integrating the corresponding diagnoses based on the symptom weights.

The Branch B assessment method achieved good results. The items “thrombophilic,” “Elderly age (≥70 years),” and “BMI >30 kg/m^2^” were calculated from the physical examination values, and the AUCs were higher than 0.95, indicating the high accuracy of these items. However, for the items “Reduced mobility” and “Ongoing hormonal treatment,” the AUC values were 0.809 and 0.827, respectively, with average prediction accuracy. Among them, the “Reduced mobility” item was more complicated to determine, and its lack of representation in the EMR may be one of the main reasons for its poor prediction. In addition, the poor matching effect of the “Ongoing hormonal treatment” item was caused by differences in doctors' judgment criteria for whether hormonal drugs were used. Taken together, although some items had average predictive effects, the overall assessment results were still reliable. In Branch B, we extracted medical data for rule determination using LAC in combination with negative word filtering. We used professional corpora, such as ICD-9-CM3, DiseaseKG, and Organization Scripted Drug List, which have wide coverage and achieve good accuracy in clinical situation determination.

Our proposed DB-DL assessment method intelligently assesses the Padua risk class of patients in Branches A and B. Branch A uses the proposed deep learning model PDCM to assess the patient's disease class. Our proposed PDCM shows the best results compared to other deep learning models, and Branch B uses a variety of professional corpora to extract and determine the clinical comprehensive factors. We achieve good accuracy with the wide coverage of the corpus we use. Ultimately, our DB-DL assessment method constructed using both A and B branches demonstrated good accuracy in assessing patients at high/low-risk levels. In addition, our method also showed high accuracy in the assessment of individual Padua table items.

In terms of practical application, the Padua scale assessment is widely used. Our proposed method is based on textual information in EMRs and only requires the extraction of diagnoses, symptoms, and other integrated medical terms from the EMR system to perform an automatic Padua risk assessment. It can be embedded in different EMR systems. We count the average time our method takes to assess a medical record, and our proposed method takes only 0.37 s on average in an EMR. The speed of human assessment of the Padua scale by doctors is mentioned in the study of Pierre et al. (10) to be ~2–14 min; our proposed method has a huge advantage compared to the speed of human assessment.

## Conclusions

In this article, we propose a dual-branch method that utilizes a deep learning model and clinically comprehensive factors to develop an intelligent method to assess the risk of VTE in patients. Compared to the doctors' assessment used as a gold standard, our proposed method attains an AUC value of 0.883 for judging high- and low-risk levels, and it takes only 0.37 s to assess an EMR. Therefore, the proposed method in this article can be applied to implement an intelligent assessment of the Padua scale and has engineering applications for assisting doctors in assessing the risk of VTE. Future studies should incorporate work that includes incorporating increasingly diverse clinical data, validating our methods in larger patient populations, and exploring advanced models and algorithms to improve assessment accuracy.

## Data availability statement

The original contributions presented in the study are included in the article/supplementary material, further inquiries can be directed to the corresponding authors.

## Ethics statement

Written informed consent was obtained from the individual(s) for the publication of any potentially identifiable images or data included in this article.

## Author contributions

JH and JY designed the study, reviewed the design and results, submitted the draft, and drafted the submitted manuscript draft. HZ provided expertise in VTE assessment and also processed and managed the data alongside JH. Additionally, JH and HZ secured funding and managed the project. All authors have read and approved the final manuscript.
